# Efficacy of Cortisone Injection in Foot and Ankle Osteoarthritis

**DOI:** 10.7759/cureus.74814

**Published:** 2024-11-30

**Authors:** Chandan noel Vincent, Jonathan Cowie, Justin Mooteeram, Hari Sugathan

**Affiliations:** 1 Trauma and Orthopaedics, Manchester Royal Infirmary, Manchester, GBR; 2 Orthopaedics and Trauma, Stepping Hill Hospital, Manchester, GBR; 3 Orthopaedics, Stepping Hill Hospital, Manchester, GBR

**Keywords:** ankle and foot, ankle osteoarthritis, image-guided injection, intra-articular steroid injections, osteoarthritis of the foot

## Abstract

Osteoarthritis (OA) of the foot and ankle is prevalent and often debilitating, necessitating effective treatment options. This study evaluates the analgesic efficacy of corticosteroid injections in individual foot and ankle joints.

Stepping Hill Hospital conducted a retrospective audit of 166 patients who received guided corticosteroid injections. We assessed pain relief using a questionnaire to obtain numerical pain scores before and after the injections.

Of the 166 patients evaluated, 51% (85) reported complete pain relief and 32% (53) reported partial relief following the injections, with a significant mean pain score reduction from 8.45 (pre-injection) to 3.42 (post-injection) (p<0.05). A total of 83 patients (47.6%) sought re-injection, which also provided significant pain relief, although slightly less effective than the first injection (pain scores: 3.42 (pre-injection) vs. 4.90 (post-injection), p<0.05). Patient satisfaction was 83% overall, with the highest satisfaction at tarsometatarsal joint injections (95%).

Corticosteroid injections for foot and ankle OA demonstrate significant analgesic benefits with high patient satisfaction. While first injections were most effective, re-injections remain a viable option, particularly for patients postponing surgery. These results support corticosteroid injections as an essential component of OA management in foot and ankle conditions.

## Introduction

Since the mid-20th century, researchers have documented the benefit of corticosteroids in treating musculoskeletal conditions, including both soft tissue and osteoarthritis (OA) [1]. Juni et al., in a Cochrane review, highlighted the importance of corticosteroid injection in the knee to relieve pain and help with functional improvements [2]. With the prevalence of foot and ankle OA thought to be as high as knee OA, the benefit of these injections needs to be considered in the smaller joints of the foot and ankle [3].

Previous studies have reviewed the efficacy of steroid injections for conditions such as Morton’s neuroma and plantar fasciitis, showing significant benefit. This study aims to review the effect on specific joints within the foot and ankle with arthritic conditions [4]. Targeted injections into affected areas could serve as a bridge to alleviate pain prior to surgery or, in some cases, eliminate the need for surgery, given the long and increasing waiting lists for surgery in the UK [5]. This study is the first to review the analgesic benefits of specific joints affected by in the foot and ankle. The study also monitored the patient's choices after experiencing a resurgence of pain following the first injection, as well as the analgesic benefits of subsequent injections.

## Materials and methods

We conducted a retrospective review of notes and a telephone questionnaire to assess the clinical outcome of all patients who received a corticosteroid injection for their foot or ankle arthritis at Stepping Hill Hospital, Stockport, in 2020. Contact was made with the patients via telephone at 4 weeks post injection and then at 6 monthly intervals. Patients were also advised to contact the orthopedic team prior to their appointment if their pain increased. Final questionnaire was performed at the end of the study at 1 year.

A consultant or senior clinical fellow in orthopedic surgery performed injections in a daycare setting using image intensifier guidance. All injections used 0.5% Marcaine with 40 mg Depo-Medrone. We excluded injections for other pathologies such as soft tissue impingement, retrocalcaneal bursitis, tenosynovitis, or Morton’s neuroma.

**Figure 1:**
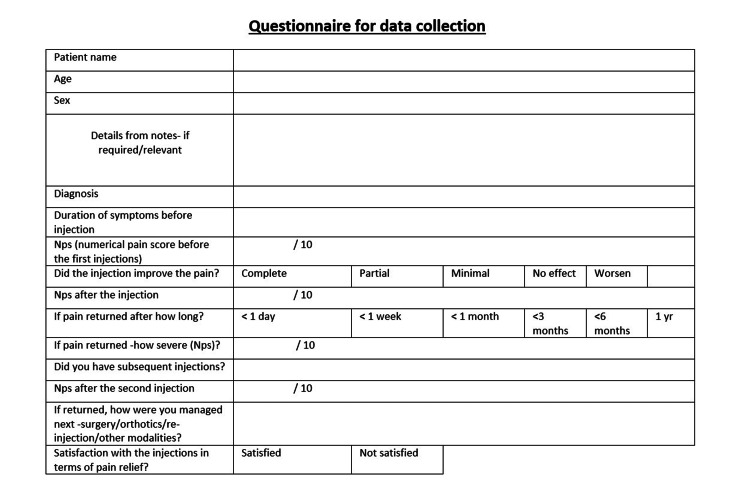
Patient questionnaire for data collection

The patients were identified from the electronic patient record databases - Bluespier, theater manager, and the patient records (case notes, letters to GP and discharge summaries). We used the hospital software, Patron/Evolve & Advantis CDS, to collect data on the diagnosis and present symptoms. Relevant imaging was assessed using picture archiving and communication system (PACS)-Sectra. We contacted the patients via telephone and obtained their verbal consent before completing the predetermined questionnaire (Figure 1). Grice et al. (2017) used a similar questionnaire in their study [4].

In 2020, we identified 177 patients who received a corticosteroid injection for of the foot or ankle. Eleven patients had passed away, and therefore the total number available for questionnaire evaluation was 166 (male: 74, female: 92). We used a numerical pain score (NPS) to assess the pre-injection and post-injection pain levels. We also assessed the duration of pain relief and documented the point of recurrence of symptoms. Subsequently, a number of patients opted for a second injection due to a recurrence of pain. We have assessed the pre-injection and post-injection pain scores of these patients to assess pain relief yielded by the second injection.

## Results

Of the 166 patients, 85 (51%) reported complete analgesic relief following the injection. A further 53 patients (32%) reported partial analgesic relief. The remaining 28 patients (16%) had little to no benefit (Table 1). No patients expressed worsening pain following a foot and ankle joint injection. There were no complications or infections from these procedures. We also assessed the duration of pain relief gained from these injections. 41 patients (25%) had a recurrence of pain before 3 months, 52 patients (31%) at 6 months post-injection, and 45 patients (27%) at the one-year mark after the first injection (Table 2).

**Table 1:** Pain relief following injection.

Pain Relief Following Injection				
Complete analgesic relief	Partial analgesic relief	Minor analgesic relief	No analgesic relief	Pain worse
85 (51%)	53 (32%)	17 (10%)	11 (7%)	0

**Table 2:** Time period between injection and pain recurrence.

Time Period of Recurrence of Pain						
Pain return time	<1 day	< 1 week	<1 month	<3 months	<6 months	<1 year
Total number	7	9	14	11	52	45
Percentage	Recurrence of pain before 3 months - 41 (25%)				31%	27%

The mean NPSs for this patient cohort were 8.45 before the injection, with a standard deviation of 1.1. Following corticosteroid injection, the NPS fell to 3.42 with a standard deviation of 2.3 (Table 3). The reduction in pain scores before and after the injection was statistically significant (p < 0.05) (Table 4).

**Table 3:** Numerical pain score (NPS) before & after first injection.

	Paired Sample Statistics			
P (<0.05)	Mean	N	Standard Deviation	Std. Error Mean
NPS before the injection	8.45	166	1.109	0.086
NPS after the injection	3.42	166	2.318	0.180

**Table 4:** Paired samples t-test before and after the first injection.

Paired Differences (P<0.05)						Significance
Numerical pain score before and after the first injection	Mean	Std Deviation	Std. Error Mean	95% Confidence Interval of the Difference		
				Lower	Upper	
	5.030	2.451	0.190	4.655	5.406	0.01

When reviewing the need for subsequent injections, 83 (47.6%) patients required further injections due to recurrence of pain. None of the patients in this data group had subsequent injections before 6 months. At 6 months post-injection, 19 (11%) had a repeat injection; this increased to a further 64 (39%) by 1 year. The mean NPS for this group before the second injection was 8.33 (Table 5). The group had a significant reduction in pain post-injection, with the average pain score being 4.90 (Table 3). The reduction in pain following a second steroid injection was statistically significant (P<0.05) (Table 6). However, comparing the average pain scores post-injection between the first (3.42) and second injection (4.90), the second injection yielded less pain relief than the first injection.

**Table 5:** Numerical pain score (NPS) before and after second injection.

	Paired Sample Statistics			
P (<0.05)	Mean	Number of Patients	Standard Deviation	Std. Error Mean
NPS before the second injection	8.47	83 (47.6%)	1.19	0.13
NPS after the second injection	4.90	83 (47.6%)	1.73	0.20

**Table 6:** Paired samples t-test before and after the second injection.

Paired Differences						Significance
Numerical pain score before and after the second injection	Mean	Std Deviation	Std. Error Mean	95% Confidence Interval of the Difference		
				Lower	Upper	
	3.567	1.88	0.205	3.254	4.071	0.01

**Figure 2:**
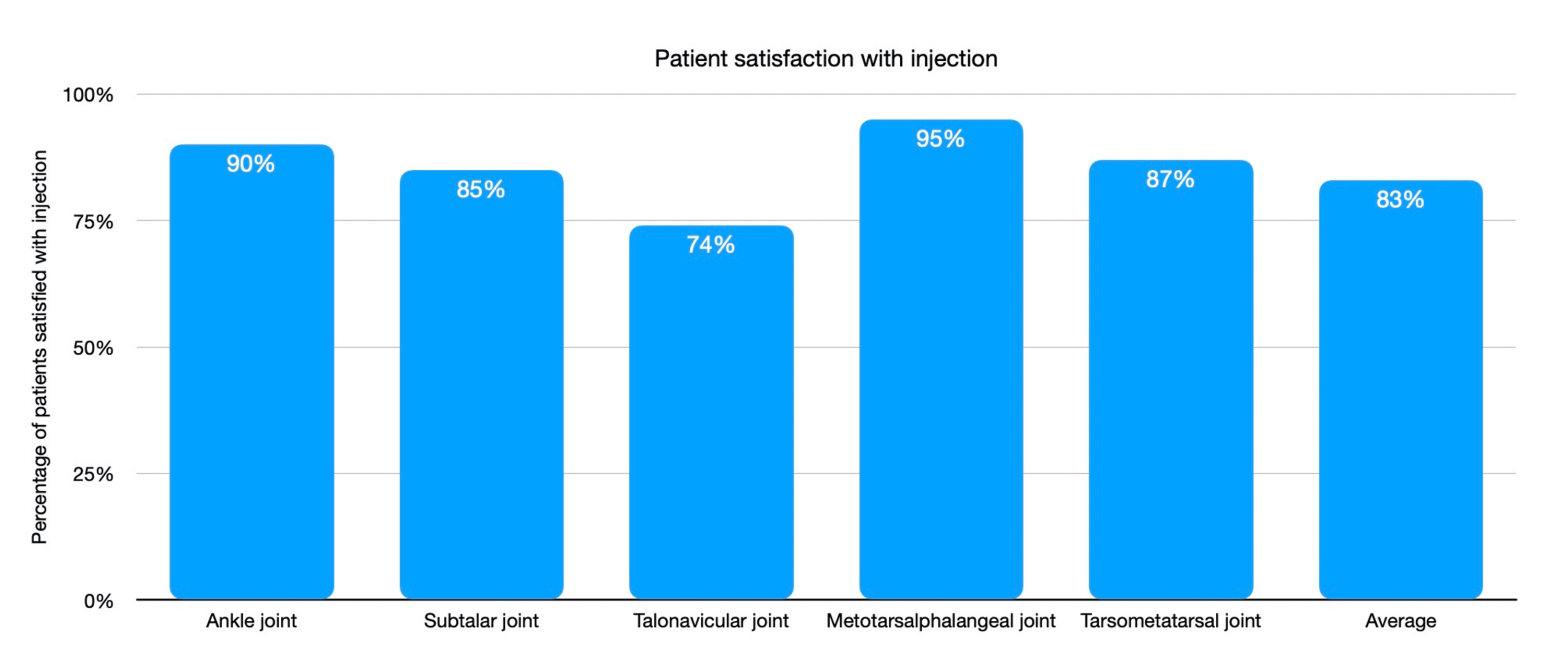
Patient satisfaction across injections in various joints

Patient satisfaction with the injection was 83% across all the groups, with the highest being in the tarsometatarsal group at 95% and the lowest in the talonavicular joint with a 74% satisfaction rate (Figure 2).

At final follow-up, when asked what option they would choose, should their pain return, 67% of patients still elected for re-injection. Twenty-three percent of patients wanted surgery as the next treatment option (Table 7). The group of patients consisted of those who were receiving ongoing analgesics, accounting for 1% of the total treatment. 

**Table 7:** Patient survey into ongoing management options.

Treatment Modality	Number of Patients	Percentage
Re-injection	111	67
Surgery	38	23
Orthotics	9	5
Analgesics	2	1
None	6	4
Total	166	100

## Discussion

Foot and ankle arthritis is a debilitating condition affecting mobility and quality of life [6]. Recent population-based studies have suggested that the prevalence of symptomatic foot and ankle arthritis within the population is 16.7% and 1.2%, respectively [7, 8]. With these figures, it is thought to be as high as of the knee, a condition regularly treated with corticosteroid injection [2]. Image-guided joint injection as a method of intervention is a well-established field for other musculoskeletal conditions [9, 10, 11]. Recently, the British Orthopaedic Foot and Ankle Society (BOFAS) and the James Lind Alliance (JLA) conducted a survey to determine future research priorities in the foot and ankle field [12]. The survey underscored the significance of investigating the effectiveness of injections for various types of foot and ankle arthritis, given the paucity of established literature in this field [12].

Our results show good resolution in pain following the injections, with average pain scores falling from 8.45 to 3.42 post-injection. The results also demonstrate recurrence of pain, which peaks at 6 months (31%-52 patients) and 1 year post injection (45 patients (27%)), with only 25% (41 patients) having recurrence before 3 months post injection. Grice et al.’s study on guided injections for various foot and ankle conditions included 365 patients who had image-guided injections. This study reported the median time to recurrence of pain as 3 months [4]. They included patients with soft tissue pathologies such as retrocalcaneal bursitis, Morton’s neuroma, and plantar fasciitis in their patient group, while the current study exclusively concentrates on arthritic conditions of the foot and ankle [4]. Ali et al. reviewed patients undergoing injections for foot and ankle arthritis and documented a similar recurrence rate, with 22% having a recurrence of symptoms within 6 months [13]. Arroll et al.’s 2004 and 2005 meta-analyses, which examined corticosteroid injections for the knee and shoulder, revealed comparable long-term positive outcomes, indicating symptom improvement at 16-24 weeks and 9 months, respectively [14, 15].

During the follow-up period, 47.6% (83 patients) opted to have a second set of injections due to a recurrence of pain. There are no studies documenting the pain relief yielded by subsequent injections and comparing its effectiveness. Grice et al., in their study, recorded 14% undergoing a further set of injections [4]. We have seen substantial pain relief yielded with the second set of injections, but when comparing average pain scores post-injections (first injection: 3.42 versus second injection: 4.90), the pain scores were comparatively lesser with the first set of injections. This suggests that subsequent foot and ankle injections may not relieve pain as well as the first. However, this could be a complex issue due to the progression of arthritis as well as systemic and local factors.

Derek et al. have evaluated the effect of corticosteroid injections for degenerative arthritis of the midfoot joints, with a significant improvement in the Self-Reported Foot and Ankle Score (SEFAS) score to 31.8 (4 months post-injection) as opposed to a SEFAS score of 17 (pre-injection). The study highlights varied responses in pain relief when comparing obese and non-obese patients. This underscores the crucial role that weight management plays in pain relief. We have not incorporated this data in our case series, but further studies reviewing both modifiable and non-modifiable risk factors have an effect on treatment efficacy [16].

The center’s retrospective review found no documented complications. Further studies report that complications from joint injections are rare but not completely undocumented [11, 17, 18, 19, 20]. The overall satisfaction following the injections was 83% across all conditions, with the lowest being for the talo-navicular joint (74%). We also assessed patient choices in case of pain recurrence, with 67% of the patients opting to have a re-injection, 23% opting for surgical intervention, and 10% are conservative (oral analgesics/orthotics). We cannot overlook the fact that some patients did not benefit from the injections, and nearly one in four still chose surgical management. Surgical management is still the likely course for the ongoing long-term management of these patients, and in most cases, corticosteroids can act as a bridging therapy to improve quality of life by reducing pain and maintaining as close to normal levels of activity as possible while awaiting definitive management. Where patients, however, do not satisfy the criteria for surgical management, making arthrodesis or arthroplasty higher risk, corticosteroid injections may act as the mainstay of treatment [21,22].

Given that the data set was gathered from a retrospective questionnaire, it is inevitable that there may be some recall bias. We could infer either positive or negative bias in this case. A senior orthopedic surgeon performed all the injections under an image intensifier; this may not be possible in certain centers due to equipment or staffing capabilities.

## Conclusions

Corticosteroids injections for foot and ankle are a safe and efficient method of therapy that improve pain and therefore quality of life for patients affected with this condition. For some patients’ analgesic benefit can last for >1 year with many other patients opting for ongoing injection as a treatment modality. Surgical management of this condition in the form of arthrodesis or arthroplasty will not be replaced by corticosteroid injection but this therapy can help in the betterment of painful symptoms. In those not fit enough for surgery it may be the only option available. The reduction in analgesic benefit in subsequent injections should be discussed with patients and ongoing long-term surgical options risk and benefits outlined.
